# *DisSetSim*: an online system for calculating similarity between disease sets

**DOI:** 10.1186/s13326-017-0140-2

**Published:** 2017-09-20

**Authors:** Yang Hu, Lingling Zhao, Zhiyan Liu, Hong Ju, Hongbo Shi, Peigang Xu, Yadong Wang, Liang Cheng

**Affiliations:** 1 Harbin Institute of Technology, School of Life Science and Technology, Harbin, 150001 People’s Republic of China; 2 Department of Computer Science and Technology, Harbin Institute of Technology, Harbin, 150001 People’s Republic of China; 3 Department of information engineering, Heilongjiang Biological Science and Technology Career Academy, Harbin, 150001 People’s Republic of China; 4 College of Bioinformatics Science and Technology, Harbin Medical University, Harbin, 150001 People’s Republic of China

**Keywords:** Functional similarity, Similarity score, Disease sets, Disease-miRNA relationships

## Abstract

**Background:**

Functional similarity between molecules results in similar phenotypes, such as diseases. Therefore, it is an effective way to reveal the function of molecules based on their induced diseases. However, the lack of a tool for obtaining the similarity score of pair-wise disease sets (SSDS) limits this type of application.

**Results:**

Here, we introduce *DisSetSim*, an online system to solve this problem in this article. Five state-of-the-art methods involving Resnik’s, Lin’s, Wang’s, PSB, and SemFunSim methods were implemented to measure the similarity score of pair-wise diseases (SSD) first. And then “pair-wise-best pairs-average” (PWBPA) method was implemented to calculated the SSDS by the SSD. The system was applied for calculating the functional similarity of miRNAs based on their induced disease sets. The results were further used to predict potential disease-miRNA relationships.

**Conclusions:**

The high area under the receiver operating characteristic curve AUC (0.9296) based on leave-one-out cross validation shows that the PWBPA method achieves a high true positive rate and a low false positive rate. The system can be accessed from http://www.bio-annotation.cn:8080/DisSetSim/.

## Background

The similarity of pair-wise disease sets (SDS) has drawn more and more attention in identifying functional similarity of the disease-caused molecules [1], predicting potential relationships between diseases and molecules [2–8], and so on. In previous studies, Wang et al. utilized the SDS to construct a human miRNA functional similarity network (MFSN) [1]. And Sun et al. used the SDS to predict novel disease lncRNA relationships [9].

The performance of calculating the SDS is mainly based on the method for computing the similarity of pair-wise diseases (SD). Currently, seven state-of-art methods involving Resnik’s [10], Lin’s [11], Wang’s [12], process-similarity based (PSB) [13], SemFunSim [14], ILNCSIM [15], and FMLNCSIM [16] methods were frequently used for computing the SD. Among these methods, Resnik’s [10], Lin’s [11], and Wang’s methods [12] are designed earlier for Gene Ontology (GO) [8, 17]. And these methods were introduced for calculating the SD by DOSim [18] and DisSim [19]. Resnik’s and Lin’s methods [10, 11] are based on information content (IC) for computing similarity between terms of ontology. And Wang’s method [12] is based on the hierarchical structure of the ontology. PSB and SemFunSim methods are newly developed for Disease Ontology (DO) [20]. PSB method [13] utilized the association of biological process between genes to calculate disease similarity. In comparison, SemFunSim method [14] considered more types of the functional associations including protein-protein interaction [21], human mRNA co-expression [22], and so on.

Resources for calculating the similarity score of pair-wise diseases (SSD) mainly includes the vocabularies of diseases and disease-related genes. The frequently used disease vocabularies contain Online Mendelian Inheritance in Man (OMIM) [23], Medical Subject Headings (MeSH) [24], and DO [20]. OMIM records the names of genetic disorders without providing semantic associations between them. MeSH provides a hierarchy of terms in biomedical domain. It contains 16 categories, of which only C and F03 involve disease names. In comparison with OMIM and MeSH, DO has been established around the concept of disease, and it aims to provide a clear definition for each disease. The disease-related genes are scattered in the databases, such as Gene Reference into Function (GeneRIF) [25], OMIM [23], Genetic Association Database (GAD) [26] and Comparative Toxicogenomics Database (CTD) [27]. It is better to use relationships of all of these databases.

“pair-wise-all pairs-maximum” (PWAPM) method and “pair-wise-best pairs-average” (PWBPA) method are optional for calculating similarity of pair-wise term sets [28]. For comparing multiple aspects, the best measure is the PWBPA method, which is widely utilized in calculating similarity of DO and GO term sets [1, 7, 9, 12].

Although DOSim [18] and DisSim [19] implemented the disease similarity methods in R package and web interface, no tools provided the function to calculate the similarity score of pair-wise disease sets (SSDS) currently. In this article, we designed and implemented an online tool *DisSetSim* to calculate the SSDS. Five state-of-art disease similarity methods (Resnik’s, Lin’s, Wang’s, PSB, and SemFunSim) and the PWBPA method was implemented in the tool. The system is freely available at http://www.bio-annotation.cn:8080/DisSetSim/.

## Methods

### Date sources

Data sets of *DisSetSim* are from open source databases, and they are listed in Table 1. DO [20] records disease names. It provides terms for calculating disease similarity. GeneRIF [25], OMIM [23], GAD [26] and CTD [27] are manually curated databases of disease-related genes. All of diseases in these databases are mapped to terms in DO based on SIDD [29]. GO annotation (GOA) [30] includes functional annotation of genes. HumanNet is the gene functional network of human. In addition, HMDD v2.0 [31] contains disease-related miRNAs, diseases of which were manually mapped to terms in DO by OAHG [32].

**Table 1:** Data sources

Data source	Web site
DO	http://disease-ontology.org/
CTD	http://ctdbase.org/
GeneRIF	http://www.ncbi.nlm.nih.gov/gene/about-generif
GAD	https://geneticassociationdb.nih.gov/
OMIM	http://www.omim.org/
GO & GOA	http://www.geneontology.org
HumanNet	http://www.functionalnet.org/humannet/
OAHG	bio-annotation.cn/OAHG/

### Methods for calculating similarity score of pair-wise diseases

Five state-of-art methods involving Resnik’s [10], Lin’s [11], Wang’s [12], PSB [13], and SemFunSim methods [14] have been implemented for calculating the SSD.

Resnik’s and Lin’s methods are based on IC. The IC of a disease *t* is described as Eq. 1:

1 $$ \mathrm{IC}\left(\mathrm{t}\right)=- lo{g}_2\frac{n_t}{N}, $$

where *N* is the total number of genes annotated by diseases, and *n*_*t*_ is the number of genes annotated by *t*. Assuming *t*_*1*_ and *t*_*2*_ are two diseases, the similarity of them is defined by Resnik as following [10]:

2 $$ {\mathrm{Sim}}_{\operatorname{Re} snik}\left({t}_1,{t}_2\right)= IC\left({t}_{MICA}\right), $$

where *t*_*MICA*_ is the most informative common ancestor (MICA) of *t*_*1*_ and *t*_*2*_. Lin defines the similarity of *t*_*1*_ and *t*_*2*_ as Eq. 3 [11]:

3 $$ {\mathrm{Sim}}_{Lin}\left({t}_1,{t}_2\right)=\frac{2\cdot IC\left({t}_{MICA}\right)}{IC\left({t}_1\right)+ IC\left({t}_2\right)}. $$

Assuming *T*_*1*_ is the set involving *t*_*1*_ and all of its ancestor terms of ontology. Semantic contribution of term *t* to *t*_*1*_ is represented as following:

4 $$ {S}_{{\mathrm{t}}_1}(t)=\left\{\begin{array}{l}1\kern13em \mathrm{t}={\mathrm{t}}_1\\ {}{S}_{{\mathrm{t}}_1}(t)=\max \left\{\mathrm{w}\cdot {S}_{{\mathrm{t}}_1}\left({t}^{\hbox{'}}\right)\kern.1em |\kern.2em {t}^{\hbox{'}}\in {T}_1\kern0.1em \right\}\kern.35em \mathrm{t}\ne {\mathrm{t}}_1\end{array}\right., $$

where *w* is the contribution factor of each semantic relationship. According to Wang et al. [1], *w* is defined as 0.5 for ‘IS_A’ relationship of DO [20]. Then, all the semantic contributions of *T*_*1*_ to *t*_*1*_ is *SV(t*_*1*_ *)*, which is defined as following:

5 $$ S\mathrm{V}\left({t}_1\right)=\sum_{\mathrm{t}\in {T}_1}{S}_{t_1}(t). $$

Assuming *T*_*2*_ is the set involving *t*_*2*_ and all of its ancestor terms, the similarity between *t*_*1*_ and *t*_*2*_ is defined as following by Wang’s method [12]:

6 $$ {\mathrm{Sim}}_{\mathrm{Wang}}\left({t}_1,{t}_2\right)=\frac{\sum_{t\in {T}_1\cap {T}_2}\left({S}_{{\mathrm{t}}_1}(t)+{S}_{t_2}(t)\right)}{SV\left({t}_1\right)+ SV\left({t}_2\right)}. $$

Assuming *t*_*1*_ and *t*_*2*_ can be related with *m* and *n* biological processes of GO based on hypergeometric test, respectively, the similarity of *t*_*1*_ and *t*_*2*_ is defined by the PSB method as following:

7 $$ {\displaystyle \begin{array}{l}{\mathrm{Sim}}_{\mathrm{PSB}}\left({t}_1,{t}_2\right)=\frac{1}{2}\Big(\frac{\sum \limits_{\mathrm{i}=1}^m\underset{1\le j\le n}{\mathit{\max \limits }}\left( Sim\left({p}_{1i},{p}_{2j}\right)\right)}{m}\\ {}\kern1.699996em +\frac{\sum \limits_{\mathrm{j}=1}^n\underset{1\le i\le m}{\mathit{\max \limits }}\left( Sim\left({p}_{2j},{p}_{1i}\right)\right)}{n}\Big)\end{array}} $$

where *p*_*1i*_ and *p*_*2j*_ is the *i*th and *j*th significant related biological process terms of *t*_*1*_ and *t*_*2*_, respectively. Sim(*p*_*1i*_, *p*_*2j*_) represents similarity between two processes *p*_*1i*_ and *p*_*2j*_, which is defined as Eq. 8:

8 $$ {\displaystyle \begin{array}{l} Sim\left({p}_1,{p}_2\right)=\frac{1}{2}\cdot \left({IC}_{GO}\left({p}_1\right)+{IC}_{GO}\left({p}_2\right)\right)\cdot \frac{n\left({p}_1\cap {p}_2\right)}{n\left({p}_1\cup {p}_2\right)}\cdot \frac{IC_{GO}\left({p}_1\right)}{\mathit{\operatorname{Max}}\left({IC}_{GO}\right)}\\ {}\cdot \frac{IC_{DO}\left({p}_1\right)}{\mathit{\operatorname{Max}}\left({IC}_{DO}\right)}\cdot \frac{IC_{GO}\left({p}_2\right)}{\mathit{\operatorname{Max}}\left({IC}_{GO}\right)}\cdot \frac{IC_{DO}\left({p}_2\right)}{\mathit{\operatorname{Max}}\left({IC}_{DO}\right)},\end{array}} $$

where *IC*_*GO*_ and *IC*_*DO*_ represent IC based on GO and DO, respectively. *n*(*p*_*1 ∩*_ *p*_*2*_) and *n*(*p*_*1 ∪*_ *p*_*2*_) denote the number of common genes of *p*_*1*_ and *p*_*2*_, and the number of total genes of *p*_*1*_ and *p*_*2*_, respectively.

Assuming *G*_*1*_ and *G*_*2*_ represent related gene sets of *t*_*1*_ and *t*_*2*_, respectively. Then, the similarity of *t*_*1*_ and *t*_*2*_ by the SemFunSim method can be described as following:

9 $$ {\displaystyle \begin{array}{l}{\mathrm{Sim}}_{\mathrm{SemFunSim}}\left({t}_1,{t}_2\right)=\frac{\sum \limits_{\mathrm{i}=1}^m\underset{1\le j\le n}{\mathit{\max \limits }}\left( Sim\left({g}_{1i},{g}_{2j}\right)\right)+\sum \limits_{j=1}^n\underset{1\le i\le m}{\mathit{\max \limits }}\left( Sim\left({g}_{2j},{g}_{1i}\right)\right)}{m+n}\\ {}\kern.999998em \cdot \frac{\mathrm{m}}{\mid {G}_{MICA}\mid}\cdot \frac{n}{\mid {G}_{MICA}\mid}\end{array}} $$

where |G_MICA_| represents the number of genes in G_MICA_. *m* and *n* denote the number of genes in *G*_*1*_ and *G*_*2*_, respectively. Sim(*g*_*1i*_, *g*_*2j*_) is the functional similarity score between genes *g*_*1i*_ and *g*_*2j*_, which could be obtained from HumanNet [33].

### Method for calculating similarity score of pair-wise disease sets

The PWBPA method was utilized for calculating the SSDS. The similarity of two disease sets *T*_*1*_ and *T*_*2*_ is defined as following:

10 $$ \mathrm{PWBPA}\left({\mathrm{T}}_1,{\mathrm{T}}_2\right)=\frac{\sum_{i=0}^N\underset{0<j\le M}{\max } Sim\left({t}_i,{t}_j\right)+\sum_{j=0}^M\underset{0<i\le N}{\max } Sim\left({t}_j,{t}_i\right)}{N+M}, $$

where *T*_*1*_ and *T*_*2*_ contains *N* and *M* diseases, respectively. *t*_*i*_ and *t*_*j*_ represents *i*th and *j*th terms of *T*_*1*_ and *T*_*2*_, respectively.

### Predicting potential association between diseases and miRNAs

Functional similarity between miRNAs could be calculated based on their related disease sets. Similarities of each pair-wise miRNAs are utilized to establish a MFSN. Node of the network represents miRNA. Weight of edge is the functional similarity score. Then, disease-related miRNAs were prioritized using the network ranking algorithm named random walk with restart (RWR) [7].

The random walker starts on one or several seed nodes and then randomly transits to neighboring nodes considering the probabilities of the edges between the two nodes. And the probability to return to the seed nodes is supposed as *γ*. Then, RWR algorithm can be defined as following:

11 $$ {\mathrm{P}}_{t+1}=\gamma {\mathrm{P}}_0+\left(1-\gamma \right){\mathrm{AP}}_t $$

where *P*_*0*_ denotes the initial probability vector, *P*_*t*_ is a vector in which the *i*th element represents the probability of finding the walker at node *i* and step *t*, *A* is the column-normalized adjacency matrix of the network. The algorithm was performed until the difference between *P*_*t*_ and *P*_*t+1*_ falling below 10^−10^, which means all the nodes become stable.

In this study, the known miRNAs of a disease were considered as seed nodes. The unknown miRNAs of it could be scored based on RWR on the MFSN. After ranking the miRNAs based on the scores, disease-related miRNAs could be prioritized.

### Implementation

**Fig. 1:**
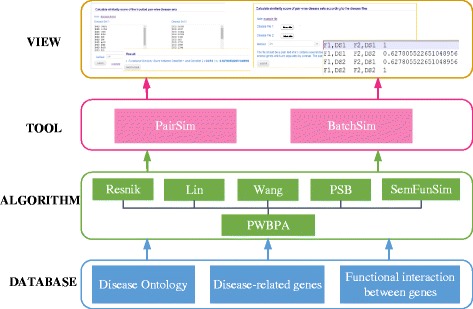
System overview of DisSetSim

*DisSetSim* has been implemented on a JavaEE framework and run on the web server (2-core (2.26 GHz) processors) of Ucloud [34]. The four-layer architecture involving DATABASE, ALGORITHM, TOOLS, and VIEW layer is shown in Fig. 1 The detailed description of the architecture is fixed as following.

(1) DATABASE layer. This layer stores DO, disease-related genes, and functional associations between genes. These are exploited by ALGORITHM layer for calculating the similarity between disease sets.

(2) ALGORITHM layer. Five algorithms of measuring the similarity between DO terms have been implemented, which include Resnik’s, Lin’s, Wang’s, PSB, and SemFunSim methods. And the method named PWBPA for calculating the SSDS were also implemented.

(3) TOOL layer. Two tools including PairSim and BatchSim have been provided for exploring the SSDS. PairSim calculates the similarity for a given pair of disease sets, and BatchSim computes similarity between each pair of multiple disease sets.

(4) VIEW layer. Web pages are provided for viewing the results. It shows the similarity of pair-wise disease sets.

## Results

### Web interface

*DisSetSim* provides two tools PairSim and BatchSim for querying the SSDS. The details about the usage of these two tools are described as follows.

#### The usage of PairSim

**Fig. 2:**
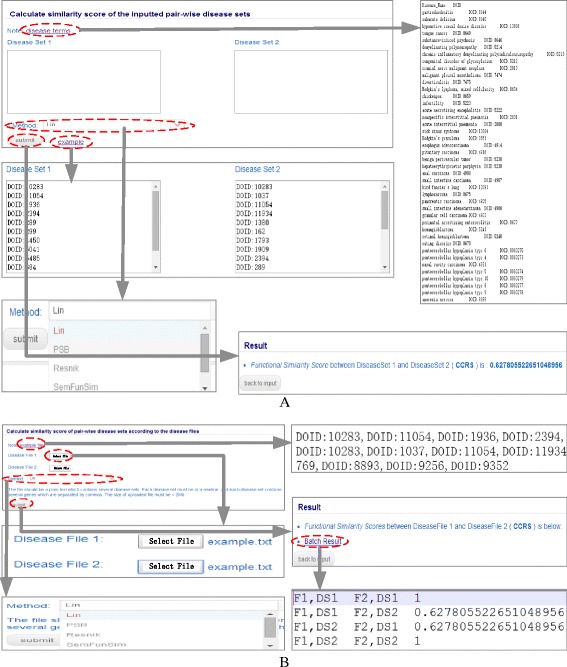
Schematic workflow of DisSetSim. **a** Schematic workflow of PairSim. **b** Schematic workflow of BatchSim

Figure 2a shows a case for searching the similarity score of a given pair of disease sets. The web page for inputting disease sets is http://www.bio-annotation.cn:8080/DisSetSim/ basic-init. Each of these disease sets could be inputted in a textbox. A disease set is comprised by several diseases. And each disease is represented by the identifier of term in DO. All the term identifiers could be downloaded from the hyperlink ‘disease terms’ in the inputting page. Here, we click the ‘example’ button to use our example. Then, we choose one of the five methods (Resnik’s, Lin’s, Wang’s, PSB, and SemFunSim) for calculating the SSD. After submitting this pair of disease sets, the system could return the similarity score based on the PWBPA method.

#### The usage of BatchSim

Figure 2b shows a case for searching the similarity score of all the pairs based on the selected files. The web page for inputting disease sets is http://www.bio-annotation.cn:8080/DisSetSim/batch-init. Two files including disease sets should be selected before submitting. The file should be a plain text which contains several disease sets. Each disease set must be in a newline, and each disease set contains several disease IDs which are separated by commas. The size of uploaded file must be <2 Mb. Here, we selected our example file in this page. Then, we choose one of the five methods for calculating the SSD. After clicking the ‘submit’ button, the system could return the similarity score of all the pairs of the selected files based on the PWBPA method.

### miRNA functional similarity network

**Fig. 3:**
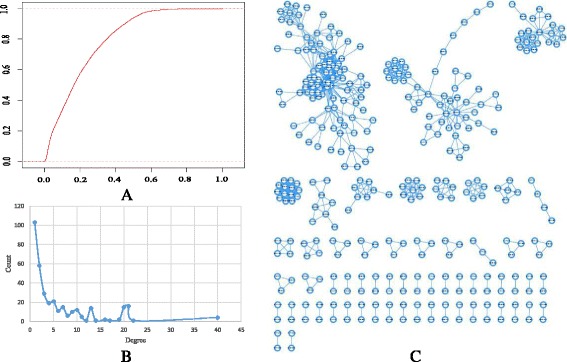
Construction and characteristics of the miRNA functional similarity network. **a** Cumulative distribution of the edges between miRNAs when using various similarity cutoffs. **b** Degree distribution for miRNA in the miRNA functional similarity network. **c** The miRNA functional similarity network

By applying *DisSetSim* to the inputted disease sets of miRNAs, the similarity score of each miRNA pair could be obtained. Using miRNA as node and similar miRNAs as edge, the MFSN was constructed based on various similarity cutoffs. As shown in Fig. 3a, the number of links dramatically decreases when the cutoff increases from low value to high value. When the cutoff is equal to or bigger than 0.7, the link numbers remain relatively stable. Therefore, we use 0.7 as cutoff for the MFSN. In total, 1042 miRNA-miRNA functional associations between 346 miRNAs were obtained as MFSN (Fig. 3c). Similar to the most of the reported biological networks, the degree of this MFSN also shows a scale-free distribution [5, 9, 35–37]. It means that most of the miRNAs only have a few functionally similar miRNAs, and a few of miRNAs have a numerous functional similar miRNA (Fig. 3b).

Here, the PWBPA method was utilized for calculating similarity between disease sets, and SemFunSim was used as computing the similarity of pair-wise diseases. This is because that the SemFunSim method was proven to obtain the best performance [14]. Alternatively, other state-of-art methods could also be chosen to construct MFSN.

### Disease-related miRNAs

**Fig. 4:**
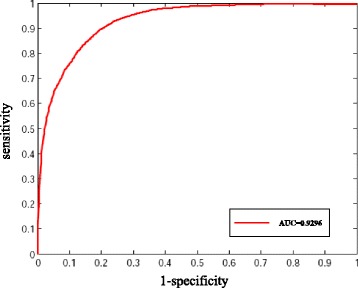
ROC curve of the PWBPA method based on leave-one-out cross validation on known experimentally verified miRNA-disease associations

By applying the above similarity scores of miRNAs, novel disease-related miRNAs were predicted based on RWR algorithm (See ‘Methods’ section). To evaluate the performance of the similarity scores of miRNAs, leave-one-out cross validation of 5710 known experimentally confirmed miRNA-disease associations, including 265 diseases with at least two miRNAs, were used for this assessment. For a disease of interest, each known miRNA of this disease was left out as the testing case, and the remaining miRNAs of this disease were used as seed nodes. All the miRNAs except the miRNAs of this disease were considered as candidate miRNAs. We then examined how well the testing miRNA ranked relative to the candidate miRNAs. If the ranking of this testing miRNA exceeded a given cutoff, we regarded this miRNA-disease association as successfully predicted. As a result, an area under the ROC curve (AUC) of 0.9296 was achieved (Fig. 4), which demonstrated that our miRNA functional similarity was effective in recovering known experimentally confirmed disease-related miRNAs.

## Discussion

As the best of our knowledge, non-coding RNAs (ncRNAs) attract more and more attentions because of their important regulation roles in molecular level. However, the lack of protein limits the identification of their function. Here the application of our tool in constructing MFSN and predicting miRNA-disease associations provides a novel way to help for exploring the function of miRNAs especially for prioritizing miRNA-disease associations. This application can be extended to other ncRNAs, such as lncRNAs and circRNAs. Although methods for calculating the SDS have been implemented by previous methods, it is not easy to calculate the SSDS. Therefore, DisSetSim benefits researchers for exploring the function of disease-related molecular.

## Conclusions

In this article, we designed and developed a web system *DisSetSim* to calculate the SSDS. Five state-of-art methods were implemented (see ‘METHODS’ section) for calculating disease similarity. And the PWBPA method was implemented for calculating the SSDS. Two tools involving PairSim and BatchSim provide the function to obtain the SSDS by inputting a pair-wise disease sets and multiple disease sets, respectively.

The functional similarity of miRNAs could be calculated based on our system. Here, the similarity of each pair-wise miRNAs was calculated. And then a MFSN was constructed based on miRNA similarity. The network was further utilized to predicate disease-related miRNAs based on RWR. The high AUC (0.9296) shows the MFSN is very suitable for predicting potential relationships between diseases and miRNAs.

## Abbreviations

CTD: Comparative Toxicogenomics Database
DO: Disease Ontology
GAD: Genetic Association Database
GeneRIF: Gene Reference into Function
GO: Gene Ontology
GOA: GO annotation
IC: information content
MFSN: miRNA functional similarity network
MICA: the most informative common ancestor
ncRNA: non-coding RNAs
OMIM: Online Mendelian Inheritance in Man
PSB: process-similarity based
PWAPM: pair-wise-all pairs-maximum
PWBPA: pair-wise-best pairs-average
PWR: random walk with restart
SD: pair-wise diseases
SSD: the similarity score of pair-wise diseases
SSDS: the similarity score of pair-wise disease sets.
